# Two-Year Follow-Up of Vascular Events in Peripheral Arterial Disease Treated With Antiplatelet Agents: A Prospective Observational Multicenter Cohort Study (SEASON)

**DOI:** 10.1038/s41598-017-06597-y

**Published:** 2017-07-21

**Authors:** Yukihito Higashi, Tetsuro Miyata, Hiroshi Shigematsu, Hideki Origasa, Masatoshi Fujita, Hiroshi Matsuo, Hiroaki Naritomi, Hiroaki Matsuda, Masahide Nakajima, Hideto Awano

**Affiliations:** 1 Department of Cardiovascular Regeneration and Medicine, Research Institute for Radiation Biology and Medicine, Hiroshima University, Hiroshima, Japan; 2 Sanno Medical Center, International University of Health and Welfare, Tokyo, Japan; 3 Division of Biostatistics and Clinical Epidemiology, University of Toyama School of Medicine, Toyama, Japan; 4 Department of Cardiovascular Medicine, Uji Hospital, Uji, Kyoto, Japan; 5 Matsuo Clinic, Osaka, Japan; 6 Senri Chuo Hospital, Osaka, Japan; 7 Mitsubishi Tanabe Pharma Corporation, Osaka, Japan

## Abstract

The present analysis was intended to evaluate the real-world management of peripheral arterial disease (PAD) in Asia, and to explore cardiovascular events in patients with PAD undergoing antiplatelet therapy over 2 years of follow-up. The Surveillance of cardiovascular Events in Antiplatelet-treated arteriosclerosis Obliterans patients in JapaN (SEASON) registry is a prospective observational multicenter study of cardiovascular events in antiplatelet-treated patients with PAD in Japan. The SEASON registry included 11,375 patients who were scheduled to receive treatment for PAD. Two analysis populations were defined: a real-world population (RWP; n = 10,322) and a definite PAD population (DPP; n = 3992) who had ankle-brachial pressure index (ABPI) <0.9 and intermittent claudication, or a history of lower limb revascularization. The primary outcome measure was the rate of the composite of cerebrovascular, cardiovascular, and peripheral vascular events. The composite event rates (95% confidence interval) were 3.28 (3.00–3.57) and 5.71 (5.13–6.34) events per 100 patient-years in the RWP and DPP groups, respectively. Fontaine IV classification and ABPI <0.4 at baseline were both identified as strong risk factors for vascular events. These findings contribute to understanding the situation for real-world patients with PAD receiving antiplatelet therapy.

## Introduction

Peripheral arterial disease (PAD) is an overall term for narrowing of the arteries other than those associated with the brain and heart, including both thromboangitis obliterans (TAO, Buerger’s disease) and atherosclerotic PAD. In Japan, there are an estimated 500,000–800,000 patients with atherosclerotic PAD, including 400,000 with symptomatic disease^1^, and 220 million people globally were estimated to have PAD in 2010, with the number of cases increasing by almost a quarter over the previous decade^2^.

The REduction of Atherothrombosis for Continued Health (REACH) registry is an international prospective observational study^3^, which enrolled approximately 68,000 patients with established vascular disease or multiple risk factors for vascular disease^4^, including 5193 patients in Japan^5^. Overall findings indicate that, while patients with PAD have a higher risk of cardiovascular events than those with coronary artery disease or cerebrovascular disease^6, 7^, improved risk factor control is associated with a positive effect on cardiovascular event rates in these patients^6^. Although baseline characteristics and 1-year outcomes have been reported for Japanese patients in the REACH registry^5, 8^, the sample size of patients with PAD was limited (n = 627). Additionally, the REACH registry only included patients with relatively severe PAD (current intermittent claudication with ankle-brachial pressure index (ABPI) <0.9 and/or a history of intermittent claudication with relevant intervention)^3^.

Oral antiplatelet agents are widely used for the treatment of patients with PAD, including patients with coldness or numbness of the lower limbs caused by occlusion of the lower arteries, which are considered to be early symptoms of PAD (Fontaine stage I)^1^. However, although supporting evidence from several national studies exists^9, 10^, direct evidence from systematic epidemiologic studies of vascular events in patients with PAD receiving antiplatelet agents is limited. Additionally, previous studies have provided conflicting reports as to whether patients with asymptomatic PAD have a better, similar, or worse prognosis than those with symptomatic PAD^11–13^.

The Surveillance of cardiovascular Events in Antiplatelet-treated arteriosclerosis Obliterans patients in JapaN (SEASON) registry was designed to provide a real-world database of over 10,000 patients with asymptomatic or symptomatic PAD treated with sarpogrelate hydrochloride or other oral antiplatelet therapy across Japan^14^. The aims of the registry were to evaluate the actual management of PAD in Japan, and to assess the incidence of cardiovascular events in patients with PAD undergoing antiplatelet therapy. Here we present the rates of vascular events over 2 years’ follow-up in the SEASON registry, and explore the impact of baseline characteristics on the risk of vascular events.

## Results

### Study Population

**Figure 1:**
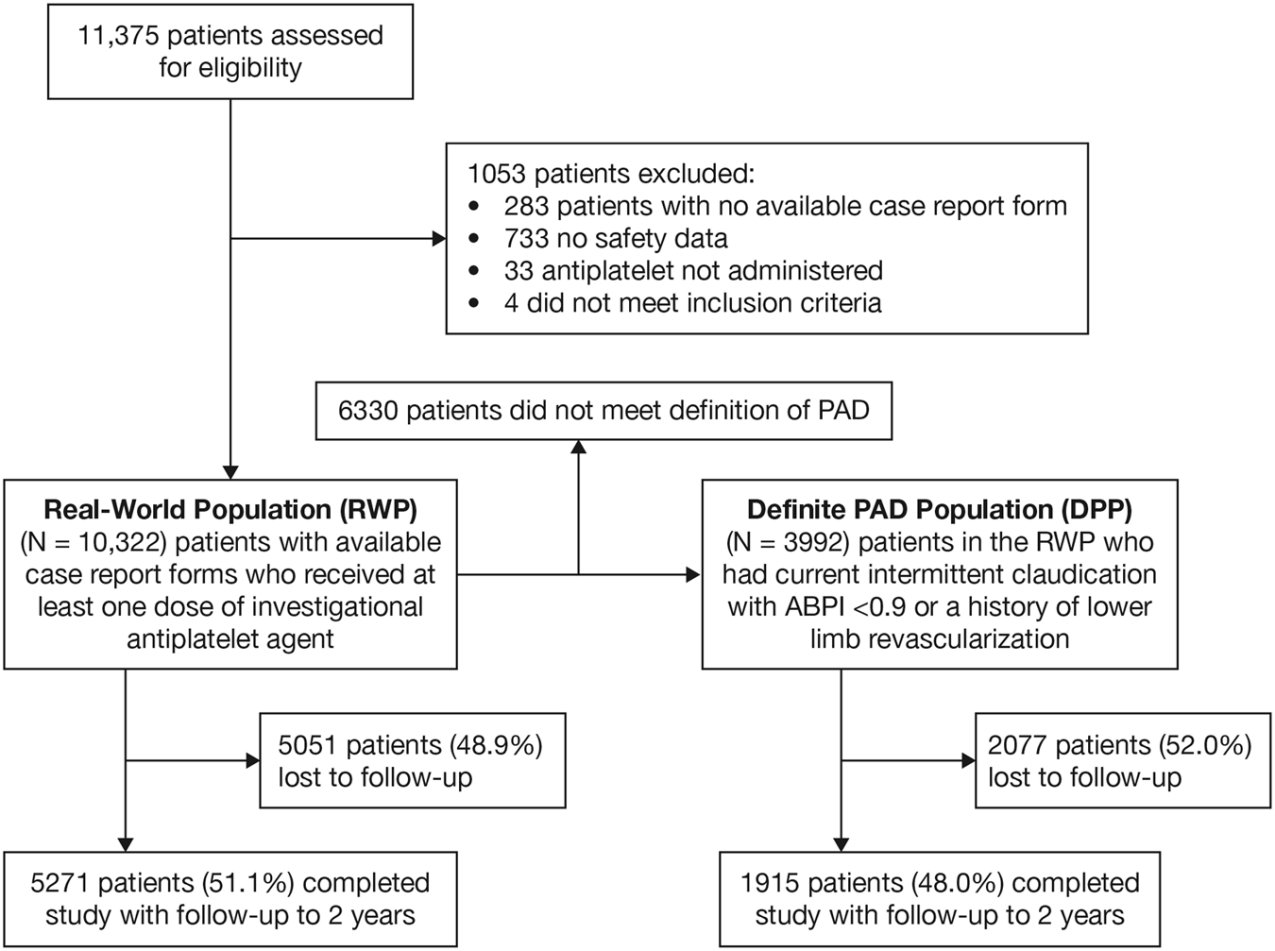
Patient Disposition. ABPI indicates ankle-brachial pressure index; PAD, peripheral arterial disease.

Patient disposition and baseline characteristics for the SEASON registry have been reported previously^15^. A total of 11,375 patients were registered at 1745 institutions. Of these, 10,322 were included in the real-world population (RWP), with a total cumulative observation time of 16,495 patient-years; the definite PAD population (DPP) included 3992 patients, with a total cumulative observation time of 6409 patient-years. Over the course of the study, 51.1% (5271/10,322) of patients in the RWP and 48.0% (1915/3992) of patients in the DPP completed 2 years of follow-up. Patient disposition during the study is shown in Fig. 1 and a summary of baseline characteristics is given in Table 1.

**Table 1:** Baseline Characteristics.

		RWP (n = 10,322) n (%)	DPP (n = 3992) n (%)
Sex	Male	6189 (60.0)	2939 (73.6)
	Female	4133 (40.0)	1053 (26.4)
Age	**Mean [SD]**	**73.8 [9.9]**	**73.5 [9.3]**
	<40	36 (0.3)	5 (0.1)
	40 s	151 (1.5)	49 (1.2)
	50 s	639 (6.2)	231 (5.8)
	60 s	2245 (21.7)	948 (23.7)
	70 s	4165 (40.4)	1644 (41.2)
	80 s	2758 (26.7)	1028 (25.8)
	≥90	328 (3.2)	87 (2.2)
BMI (kg/m^2^)	**Mean [SD]**	**22.81 [3.66]**	**22.49 [3.47]**
	<25.0	6951 (67.3)	2967 (74.3)
	25.0–29.9	1902 (18.4)	710 (17.8)
	30.0–34.9	258 (2.5)	86 (2.2)
	≥35.0	51 (0.5)	7 (0.2)
	Unknown	1160 (11.2)	222 (5.6)
Fontaine Classification	Stage I	4215 (40.8)	414 (10.4)
	Stage II	4445 (43.1)	2778 (69.6)
	Stage III	1090 (10.6)	412 (10.3)
	Stage IV	570 (5.5)	387 (9.7)
	Unknown	2 (0.0)	1 (0.0)
ABPI	<0.40	331 (3.2)	303 (7.6)
	0.40–0.69	1953 (18.9)	1680 (42.1)
	0.70–0.89	2205 (21.4)	1515 (38.0)
	0.90–0.99	776 (7.5)	117 (2.9)
	1.00–1.39	1296 (12.6)	132 (3.3)
	≥1.40	4 (0.0)	0
	Unknown	3757 (36.4)	245 (6.1)
History of Limb Revascularization	Without	8885 (86.1)	2597 (65.1)
	With	1387 (13.4)	1387 (34.7)
	Unknown	50 (0.5)	8 (0.2)
History of Limb Amputation	Without	10,117 (98.0)	3788 (94.9)
	With	202 (2.0)	202 (5.1)
	Unknown	3 (0.0)	2 (0.1)
Smoking History	Never smoked	4445 (43.1)	1120 (28.1)
	Past smoker	2594 (25.1)	1471 (36.8)
	Current smoker	1677 (16.2)	888 (22.2)
	Unknown	1606 (15.6)	513 (12.9)
History/Complication	Hypertension	6508 (63.0)	2800 (70.1)
	Dyslipidemia	4170 (40.4)	1814 (45.4)
	Diabetes mellitus	3953 (38.3)	1805 (45.2)
	Chronic kidney disease	1473 (14.3)	734 (18.4)
	Heart disease	3064 (29.7)	1546 (38.7)
	Cerebrovascular disease	1767 (17.1)	779 (19.5)
Medication	Sarpogrelate	8416 (81.5)	3262 (81.7)
Combined Medications	Aspirin	2450 (23.7)	1468 (36.8)
	Antiplatelets other than aspirin	1573 (15.2)	788 (19.7)
	Statin	3164 (30.7)	1427 (35.7)

Abbreviations: ABPI, ankle-brachial pressure index; BMI, body mass index; DPP, definite peripheral arterial disease population; RWP, real-world population; SD, standard deviation.

### Vascular Events

A summary of all vascular events reported to the EERC and the number of these judged to be included as SEASON events are shown in Supplementary Table 1. The rate (95% CI) for the combined endpoint of any cerebrovascular, cardiovascular, or peripheral vascular event was 3.28 (3.00–3.57) per 100 patient-years in the RWP and 5.71 (5.13–6.34) per 100 patient-years in the DPP. Observed rates of the composite endpoints for cerebrovascular, cardiovascular, and peripheral vascular events and individual endpoints within each category in the RWP and DPP are shown in Table 2.

**Table 2:** Vascular Event and Mortality Rates Over 2 Years (RWP and DPP).

Event Category	RWP (n = 10,322)			DPP (n = 3992)		
	No. of Events	Risk Rate (events per 100 patient-years)	95% CI	No. of Events	Risk Rate (events per 100 patient-years)	95% CI
**Composite Event**	**528**	**3.28**	**3.00–3.57**	**351**	**5.71**	**5.13–6.34**
**Cerebrovascular Event**	**175**	**1.07**	**0.92–1.24**	**104**	**1.64**	**1.34–1.98**
Cerebral infarction	128	0.78	0.65–0.93	82	1.29	1.03–1.60
Intracerebral hemorrhage	24	0.15	0.09–0.22	12	0.19	0.10–0.33
Subarachnoid hemorrhage	9	0.05	0.02–0.10	5	0.08	0.03–0.18
Transient ischemic attack	16	0.10	0.06–0.16	7	0.11	0.04–0.23
**Cardiovascular Event**	**206**	**1.26**	**1.09–1.45**	**131**	**2.08**	**1.74–2.47**
Myocardial infarction	48	0.29	0.21–0.39	31	0.49	0.33–0.69
Unstable angina	48	0.29	0.22–0.39	31	0.49	0.33–0.69
Heart failure	117	0.71	0.59–0.85	72	1.13	0.89–1.43
**Peripheral Vascular Event**	**180**	**1.10**	**0.95–1.27**	**139**	**2.21**	**1.86–2.61**
Amputation	70	0.43	0.33–0.54	59	0.93	0.71–1.20
Development of critical limb ischemia	38	0.23	0.16–0.32	31	0.49	0.33–0.69
Acute limb ischemia	54	0.33	0.25–0.43	45	0.71	0.52–0.95
New onset of end-stage renal failure	17	0.10	0.06–0.17	8	0.12	0.05–0.25
Acute aortic dissection	4	0.02	0.01–0.06	0	0.00	0.00–0.00
Rupture of abdominal aortic aneurysm	5	0.03	0.01–0.07	2	0.03	0.00–0.11
Acute pulmonary thromboembolism	1	0.01	0.00–0.03	0	0.00	0.00–0.00
**Fatal SEASON Event**	84	0.51	0.41–0.63	44	0.69	0.50–0.92
Fatal cerebrovascular event	31	0.19	0.13–0.27	20	0.31	0.19–0.48
Fatal cardiovascular event	40	0.24	0.17–0.33	21	0.33	0.20–0.50
Fatal peripheral vascular event	13	0.08	0.04–0.13	3	0.05	0.01–0.14
**Death From Any Cause**	520	3.15	2.89–3.44	249	3.89	3.42–4.40
**Any SEASON Event or Death From Any Cause**	922	5.72	5.36–6.10	528	8.59	7.88–9.36

Abbreviations: CI, confidence interval; DPP, definite peripheral arterial disease population; RWP, real-world population.

### Mortality

During the course of the study, there were 520 deaths from any cause in the RWP, and 249 deaths in the DPP. Of these, there were 84 deaths (risk rate: 0.51 [0.41–0.63] deaths per 100 patient-years) related to SEASON events in the RWP during the study, and 44 deaths (risk rate: 0.69 [0.50–0.92] deaths per 100 patient-years) in the DPP. A summary of deaths attributed to SEASON events is shown in Table 2.

**Figure 2:**
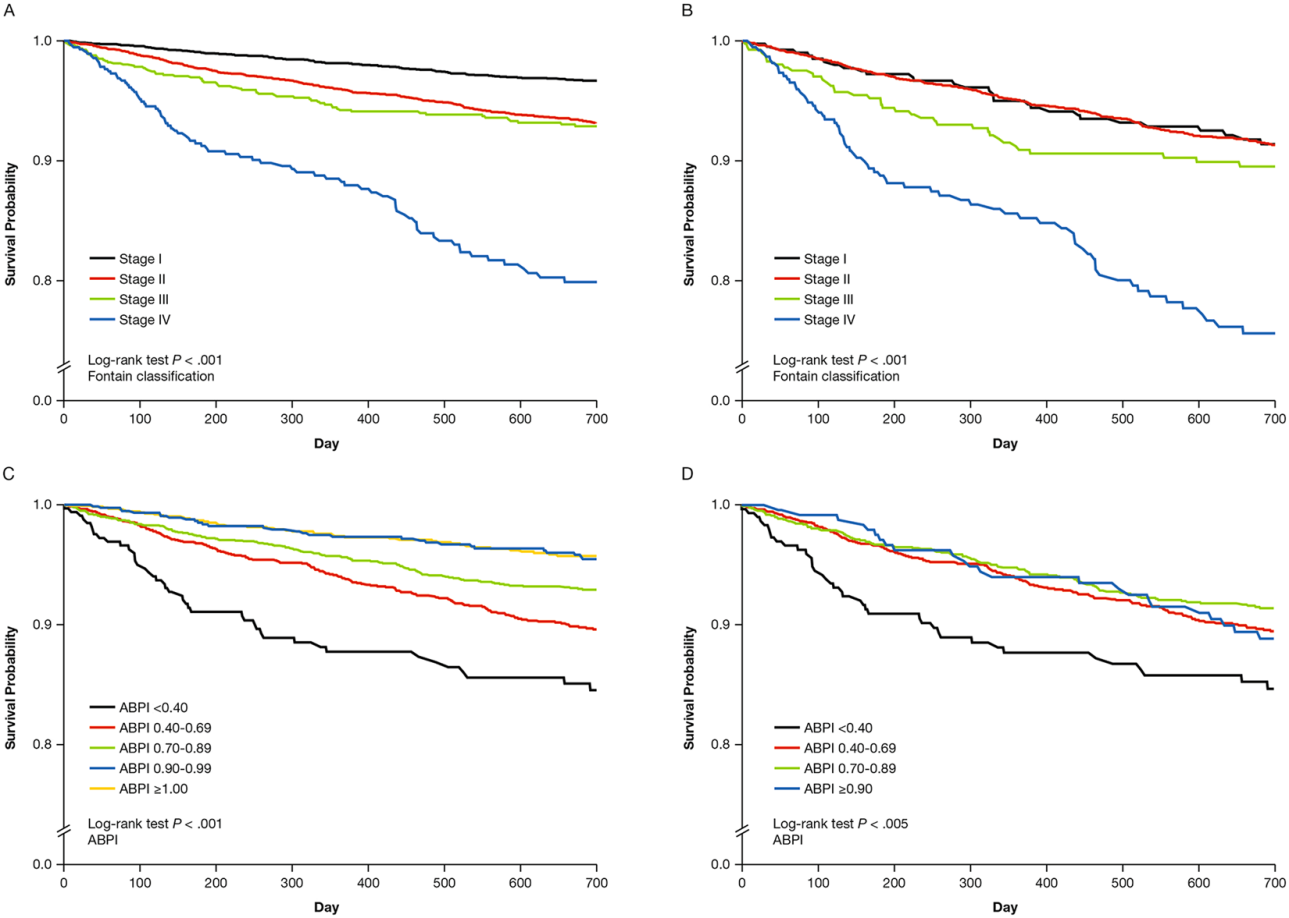
Kaplan-Meier Curves for Composite Events Over 2 Years (RWP and DPP) Composite events in (**A)** the RWP by Fontaine classification at baseline, (**B)** the DPP by Fontaine classification at baseline, (**C)** the RWP by ABPI classification at baseline, and (**D)** the DPP by ABPI classification at baseline.

In a post hoc analysis, among patients in the RWP, the rate of any SEASON event or death from any cause was 5.72 (95% CI, 5.36–6.10) per 100 patient-years, compared with 8.59 (95% CI, 7.88–9.36) in the DPP group, with a 2-year cumulative event-free survival rate of 89.55% in the RWP, and 84.83% in the DPP. Kaplan-Meier curves of the composite event in the RWP and DPP according to Fontaine classification and ABPI are shown in Fig. 2.

A summary of adjusted hazard ratios for the composite event by baseline subgroup in the RWP and DPP are presented in Table 3. In both the RWP and the DPP groups, severe Fontaine classification, abnormal ABPI, chronic kidney disease, current smoking, heart disease, and diabetes mellitus history at baseline were all associated with a higher risk of experiencing vascular events.

**Table 3:** Adjusted Hazard Ratios Based on Time to the Composite Event by Baseline Covariates and Treatment.

		RWP		DPP	
		Hazard Ratio^a^	95% CI	Hazard Ratio^a^	95% CI
Sex	Male	1.07	0.81–1.41	0.98	0.72–1.35
Age	60–69	1.23	0.76–2.01	1.31	0.75–2.29
	70–79	1.38	0.85–2.21	1.44	0.83–2.50
	≥80	1.37	0.83–2.26	1.42	0.80–2.53
BMI	<18.5	1.40	0.95–2.07	1.25	0.79–1.99
	18.5–24.9	1.32	1.01–1.74	1.38	1.01–1.90
Fontaine Classification	Stage II	1.49	1.13–1.98	0.93	0.61–1.42
	Stage III	1.64	1.09–2.46	0.85	0.49–1.49
	Stage IV	4.22	2.92–6.10	2.86	1.76–4.64
ABPI^b^	0.99–0.90	1.01	0.61–1.67	—	—
	0.89–0.70	1.36	0.93–2.00	0.79	0.48–1.28
	0.69–0.40	1.58	1.07–2.34	0.84	0.52–1.36
	<0.40	2.42	1.51–3.88	1.29	0.74–2.24
Smoking	Past smoker	1.09	0.82–1.45	0.97	0.70–1.35
	Current smoker	1.45	1.06–1.99	1.47	1.03–2.09
History/Complication	Hypertension	1.20	0.91–1.58	1.13	0.83–1.54
	Diabetes mellitus	1.31	1.06–1.63	1.35	1.06–1.73
	Chronic kidney disease	2.00	1.59–2.52	1.86	1.43–2.43
	Heart disease	1.41	1.12–1.78	1.29	0.99–1.68
	Cerebrovascular disease	1.18	0.92–1.51	1.22	0.93–1.62
Medication	Sarpogrelate	0.93	0.71–1.21	0.98	0.72–1.32
Combined Medications	Aspirin	0.92	0.73–1.16	0.83	0.64–1.08
	Antiplatelets other than aspirin	1.11	0.89–1.39	1.09	0.85–1.39
	Statin	1.02	0.82–1.27	1.07	0.83–1.38

Abbreviations: ABPI, ankle-brachial pressure index; BMI, body mass index; CI, confidence interval; DPP, definite peripheral arterial disease population; RWP, real-world population.

^a^Hazard ratio adjusted for sex, age, BMI, Fontaine classification, ABPI, smoking, history/complication (hypertension, diabetes mellitus, chronic kidney disease, heart disease, cerebrovascular disease), medication (sarpogrelate), combined medications (aspirin agents, other antiplatelet agent except aspirin, statin).

References for hazard ratios: sex (female), age (under 60), BMI (≥25), Fontaine classification (Stage I), ABPI (≥1.0 for RWP and ≥0.90 for DPP), smoking (non-smoker), history/complication (without each history/complication), medication (cilostazol, ticlopidine, prostaglandin I_2_, prostaglandin E_1_, or eicosapentaenoic acid), combined medications (without each agent).

^**b**^Compared with ABPI ≥1.00 in the RWP and compared with ABPI ≥0.90 in DPP.

## Discussion

The SEASON registry included Japanese patients with asymptomatic or symptomatic PAD undergoing antiplatelet therapy, providing the first large observational prospective database to enable epidemiologic analysis of vascular events in this population. These findings contribute to our understanding of the real-world situation for patients with PAD receiving antiplatelet therapy seen in clinical practice in Japan.

In both the RWP, which included patients with both asymptomatic and symptomatic PAD, and the DPP, the subpopulation of RWP patients with more confirmed symptomatic PAD, the most common cerebrovascular, cardiovascular, and peripheral vascular events that occurred were cerebral infarction, heart failure, and amputation, respectively. Over 2 years, the rate of any cerebrovascular, cardiovascular, or peripheral vascular event observed in the SEASON registry was numerically lower in the RWP compared with the DPP, with numerically lower incidence rates for each of the individual composite outcomes. Rates of fatal vascular, cerebrovascular, and cardiovascular events, and all causes of death were also numerically lower in the RWP compared with the DPP, although conversely, rates of fatal peripheral vascular events were numerically greater in the RWP. Furthermore, event-free survival at 2 years was numerically greater in the RWP compared with the DPP. Overall, these findings are consistent with a worse prognosis for patients with more severe PAD, as in the DPP population.

In the Japanese cohort of the REACH registry, which included only patients with relatively severe PAD^8^, the 1-year incidence of nonfatal myocardial infarction stroke, or death from cardiovascular causes, was 3.08 events per 100 patient-years, numerically higher than the 2.45 events per 100 patient-years observed when summing these three events for the DPP in SEASON. While the observed incidence of cardiovascular-related death alone in the REACH and SEASON registries were similar (0.55 vs 0.69 events per 100 patient-years, respectively)^15, 16^, there were greater numerical differences between the observed incidence of myocardial infarction and stroke (0.77 vs 0.49 events per 100 patient-years and 2.07 vs 1.29 events per 100 patient-years, respectively). However, while there are significant similarities between the patient populations included in the SEASON and REACH registries^15, 16^, any direct comparison must be interpreted with caution, owing to differences in the definitions of key outcomes, such as stroke, used in each study. Furthermore, data were reported differently in the two studies, with the SEASON study analysis being based on physician-reported events that were evaluated and classified as SEASON events by the EERC, while the REACH study used reported events directly.

In the regression analysis to identify prognostic factors, both Fontaine IV classification and ABPI <0.4 at baseline were strongly associated with a greater risk of experiencing vascular events over the 2-year follow-up period. These and the other risk factors identified in the regression analysis were generally consistent with those reported in previous studies^17–19^.

Although the large population sizes associated with a nationwide registry contribute to the strength of the analysis, there are several limitations associated with observational cohort studies such as SEASON^14, 15^. Only centers that prescribed sarpogrelate were eligible for inclusion in the registry, and patients at these centers were not recruited sequentially, with a fixed sampling ratio of patients who received sarpogrelate to other antiplatelet agents, introducing potential sampling bias. As only patients who were scheduled to receive antiplatelet therapy were eligible for inclusion, no data from healthy subjects or patients who did not have PAD, or from patients with PAD who did not receive antiplatelet treatment, were obtained for comparison or for assessment of the incidence of PAD itself. Finally, as is common to longitudinal observational studies where all follow-up data is collected by physicians, approximately 50% of the study population were lost to follow-up over the course of the study. This is in contrast to studies where follow-up is done by telephone survey through co-medicals, where a higher follow-up rate could be expected. Inclusion of the data relating to those patients lost to follow-up, if it had been available, could have significantly affected the findings of this analyses.

The large sample size included in this study suggests that the findings presented here are likely to be broadly representative of the expected outcomes for Japanese patients with PAD receiving antiplatelet therapy. Furthermore, the estimated cumulative event-free survival rates at 2 years were 0.90 in the RWP and 0.85 in the DPP, in line with those reported in a German study at 2 years for the asymptomatic and symptomatic PAD populations, respectively; thus, the findings presented here may also be applicable to other similar populations worldwide^12^.

The prevalence of acute myocardial infraction (0.49 events per 100 patient-years) was very low in this study population. It has been shown that six Japanese cohort populations have the lowest prevalence of acute myocardial infraction among other countries registered in World Heath Organization Multinational Monitoring of Trends and Determinants in Cardiovascular Disease Project, and the prevalence of acute myocardial infraction in Japanese populations is only approximately one-tenth to one-fifteenth of that in Caucasian populations^20, 21^. However, the reasons for the very low incidence of acute myocardial infraction in Japanese populations remain unclear.

## Conclusions

The large observational prospective SEASON registry has demonstrated vascular event rates over 2 years in Japanese patients with PAD undergoing antiplatelet treatment. Risk factors for vascular events were consistent with those seen in other international populations, including Fontaine IV classification and ABPI <0.4 at baseline, which were both identified as strong risk factors for vascular events. These findings contribute to the understanding of the real-world situation of patients with PAD receiving antiplatelet therapy in Japan.

## Methods

### Study Design

The SEASON registry was an observational prospective cohort study conducted across multiple sites in Japan in accordance with the Good Postmarketing Study Practice guidelines as specified by the Ministry of Health, Labour and Welfare. Participation in the study was restricted to sites that prescribed sarpogrelate. Patients were enrolled at participating sites between September 2009 and September 2011, and followed for up to 2 years. The protocol was registered in the University hospital Medical Information Network (UMIN) Clinical Trials Registry (UMIN000003385). Full details of the design and conduct of SEASON have been published previously^14, 15^.

### Study Population

The study included patients with a confirmed clinical diagnosis of PAD who were scheduled to receive long-term oral antiplatelet therapy with sarpogrelate, cilostazol, ticlopidine, prostaglandin I_2_ (beraprost sodium), prostaglandin E_1_ (limaprost alfadex), or eicosapentaenoic acid, or modifying existing long-term antiplatelet therapy by switching to or adding one of the designated antiplatelet agents. However, patients were excluded if they were already receiving sarpogrelate at the time of registration. Two analysis populations were defined: a RWP comprising patients with available case report forms who received at least one dose of investigational antiplatelet agent, and a DPP comprising a subpopulation of patients in the RWP who had current intermittent claudication with an ABPI <0.9 or a history of lower limb revascularization. All methods were carried out in accordance with relevant guidelines and regulations. The study protocol was approved by the Ethics Review Board of Hiroshima University, International University of Health and Welfare, University of Toyama School of Medicine, Uji Hospital, Matsuo Clinic, and Senri Chuo Hospital. All patients received an explanation about the study before the enrollment. Informed concent was obtained from all subjects.

### Study Assessments

Baseline assessments included demographics, premedication, current concomitant medications, medical history of PAD, severity of ischemic symptoms, physical examination, laboratory values, and risk factors/comorbidities. All follow-up data were collected directly from outpatients by physicians. Vascular events and changes in ischemic symptoms were evaluated every 6 months over 2 years; all vascular events were reported to the Efficacy Endpoint Review Committee (EERC) at the time of onset. The Committee assessed the appropriateness of the clinical judgment of vascular events according to prespecified criteria, with members of the Committee blinded with respect to antiplatelet treatment (prospective randomized open-blinded end-point method)^22^. The EERC could request that physicians provide additional clinical information for assessment, and any differences in opinion under assessment were resolved by discussion. All events judged by the EERC to be cerebrovascular events (cerebral infarction, intracranial hemorrhage, subarachnoid hemorrhage, and transient ischemic attack), cardiovascular events (acute myocardial infarction, unstable angina, and heart failure) or peripheral vascular events (amputation, development of critical limb ischemia, acute limb ischemia, acute aortic dissection, rupture of an abdominal aortic aneurysm, acute pulmonary thromboembolism, and new-onset end-stage renal failure) were included as outcome events (SEASON events) in the analysis. Definition of amputation included minor and major amputations. Major amputation is defined as above the ankle amputation. Amputations caused by a tumor or trauma were excluded. In cases where two or more qualifying events were recorded for an individual patient, only the first incident was included for the purpose of the analysis.

### Outcomes

The primary endpoint was the rate of SEASON events (a composite of cerebrovascular, cardiovascular, and peripheral vascular events, as confirmed by the EERC) per 100 patient-years. Secondary endpoints included: rates per 100 patient-years for composite cerebrovascular, cardiovascular, and peripheral vascular events as separate groups; deaths relating to any SEASON event; cerebrovascular deaths; cardiovascular deaths; peripheral vascular deaths; and death from any cause. An exploratory analysis was also conducted to examine the relationship between patient characteristics at baseline and the primary endpoint.

### Sample Size

The rationale for the planned sample size of approximately 10,000 patients, consisting of 8000 patients initiating sarpogrelate and 2000 initiating one of the other designated antiplatelet agents, has been described previously^14^.

### Statistical Analyses

The mean, median, standard deviation, and range for continuous data and counts or percentages for categorical data were calculated. Event rates per 100 patient-years for the primary and secondary endpoints were calculated by dividing the total number of patients with events at 2 years by the total duration (years) of patient participation in the registry, multiplied by 100, with 95% confidence intervals (CIs) calculated based on the assumption of a Poisson distribution. For patients who failed to attend a scheduled visit and were lost to follow-up, the previous visit was counted as the last day and used to calculate the observed period. All data until ‘lost to follow-up’ were included in the total observed period, and the events that occurred within the period were included in calculation of the endpoints.

Event rates for the primary endpoint were calculated for baseline subgroups and Kaplan-Meier curves were estimated, with the log-rank test performed to detect differences between subgroups within each variable.

Cox regression was performed to identify predictors of 2-year composite cerebrovascular, cardiovascular, and peripheral vascular events. Variables included in the Cox model were patient characteristics including sex, age, body mass index, Fontaine classification, ABPI; smoking history; risk factors including hypertension, diabetes mellitus, chronic kidney disease, heart disease, cerebrovascular disease; and antiplatelet treatment, including sarpogrelate or other antiplatelet agents. No imputation method was used with regard to missing values. The rate of any SEASON event or death from any cause was also calculated for comparison with other published reports as part of an ad-hoc analysis.

Two-tailed *P* values were calculated using a cut-off of .05 for significance, and two-sided CIs were calculated. All analyses were performed using SAS version 9.3 (SAS Institute, Cary, NC, USA).

### Clinical Trial Registration Information

Surveillance of cardiovascular Events in Antiplatelet-treated arteriosclerosis Obliterans patients in JapaN (SEASON) registry (http://www.umin.ac.jp/ctr: UMIN000003385).

## Electronic supplementary material

Supplementary Information 

## References

[1] Shigematsu H, Ikeda Y, Ishimaru S (2009). Guidelines for the management of peripheral arterial occlusive diseases. Circ J.

[2] Fowkes FG (2013). Comparison of global estimates of prevalence and risk factors for peripheral artery disease in 2000 and 2010: a systematic review and analysis. Lancet.

[3] Ohman EM (2006). REACH Registry Investigators. The REduction of Atherothrombosis for Continued Health (REACH) Registry: an international, prospective, observational investigation in subjects at risk for atherothrombotic events-study design. Am Heart J.

[4] Bhatt DL (2006). REACH Registry Investigators. International prevalence, recognition, and treatment of cardiovascular risk factors in outpatients with atherothrombosis. JAMA.

[5] Yamazaki T (2007). REACH Registry Investigators. Prevalence, awareness and treatment of cardiovascular risk factors in patients at high risk of atherothrombosis in Japan. Circ J.

[6] Alberts MJ (2009). REduction of Atherothrombosis for Continued Health Registry Investigators. Three-year follow-up and event rates in the international REduction of Atherothrombosis for Continued Health Registry. Eur Heart J.

[7] Steg PG (2007). REACH Registry Investigators. One-year cardiovascular event rates in outpatients with atherothrombosis. JAMA.

[8] Uchiyama S (2009). REduction of Atherothrombosis for Continued Health Registry Investigators. Cardiovascular event rates in patients with cerebrovascular disease and atherothrombosis at other vascular locations: results from 1-year outcomes in the Japanese REACH Registry. J Neurol Sci.

[9] Gotoh F (2000). Cilostazol stroke prevention study: a placebo-controlled double-blind trial for secondary prevention of cerebral infarction. J Stroke Cerebrovasc Dis.

[10] Origasa H, Ikeda Y, Shimada K, Shigematsu H (2004). Oral beraprost sodium as a prostaglandin I_2_ analogue for vascular events in patients with peripheral arterial disease: meta-analysis of two placebo-controlled randomized trials. Jpn J Pharmacoepidemiol.

[11] Criqui MH (1992). Mortality over a period of 10 years in patients with peripheral arterial disease. N Engl J Med.

[12] Diehm C (2009). German Epidemiological Trial on Ankle Brachial Index Study Group. Mortality and vascular morbidity in older adults with asymptomatic versus symptomatic peripheral artery disease. Circulation.

[13] Leng GC (1996). Use of ankle brachial pressure index to predict cardiovascular events and death: a cohort study. BMJ.

[14] Higashi Y (2010). SEASON Investigators. Study design of SEASON registry: prospective Surveillance of cardiovascular Events in an Antiplatelet-treated arterioSclerosis Obliterans patients in JapaN (SEASON). Int Heart J.

[15] Higashi Y (2016). SEASON Investigators. Baseline characterization of Japanese peripheral arterial disease patients - analysis of Surveillance of cardiovascular Events in an Antiplatelet-treated arterioSclerosis Obliterans patients in JapaN (SEASON). Circ J.

[16] Krempf M (2010). Cardiovascular event rates in diabetic and nondiabetic individuals with and without established atherothrombosis (from the REduction of Atherothrombosis for Continued Health [REACH] Registry). Am J Cardiol.

[17] Bhatt DL (2010). REACH Registry Investigators. Comparative determinants of 4-year cardiovascular event rates in stable outpatients at risk of or with atherothrombosis. JAMA.

[18] Howard DP (2015). Population-based study of incidence, risk factors, outcome, and prognosis of ischemic peripheral arterial events: implications for prevention. Circulation.

[19] Rac-Albu M, Iliuta L, Guberna SM, Sinescu C (2014). The role of ankle-brachial index for predicting peripheral arterial disease. Maedica (Buchar).

[20] Tunstall-Pedoe H (1994). Myocardial infarction and coronary deaths in the World Health Organization MONICA Project. Registration procedures, event rates, and case-fatality rates in 38 populations from 21 countries in four continents. Circulation.

[21] Ueshima H (2007). Explanation for the Japanese paradox: prevention of increase in coronary heart disease and reduction in stroke. J Atheroscler Thromb.

[22] Hansson L, Hedner T, Dahlof B (1992). Prospective randomized open blinded end-point (PROBE) study. A novel design for intervention trials. Prospective Randomized Open Blinded End-Point. Blood Press.

