# Supplementary material for: Two-Year Follow-Up of Vascular Events in Peripheral Arterial Disease Treated With Antiplatelet Agents: A Prospective Observational Multicenter Cohort Study (SEASON)
Source: Sci Rep. 2017 Jul 21;7:6095. doi: 10.1038/s41598-017-06597-y (PMC5522420; doi:10.1038/s41598-017-06597-y)
Supplement: Supplementary file 1 — Supplementary Information [file 41598_2017_6597_MOESM1_ESM.pdf]

Supplementary Data

Two-Year Follow-Up of Vascular Events in Peripheral Arterial Disease Treated With Antiplatelet Agents: A Prospective Observational Multicenter Cohort Study (SEASON)

Yukihito Higashi, MD[1]; Tetsuro Miyata, MD[2]; Hiroshi Shigematsu, MD[2]; Hideki Origasa, PhD[3]; Masatoshi Fujita, MD[4]; Hiroshi Matsuo, MD[5]; Hiroaki Naritomi, MD[6]; Hiroaki Matsuda, MPH[7]; Masahide Nakajima, PhD[7]; and Hideto Awano, MS[7], for the SEASON Investigators

1 Department of Cardiovascular Regeneration and Medicine, Research Institute for Radiation Biology and Medicine, Hiroshima University, Hiroshima, Japan
2 Sanno Medical Center, International University of Health and Welfare, Tokyo, Japan
3 Division of Biostatistics and Clinical Epidemiology, University of Toyama School of Medicine, Toyama, Japan
4 Department of Cardiovascular Medicine, Uji Hospital, Uji, Kyoto, Japan
5 Matsuo Clinic, Osaka, Japan
6 Senri Chuo Hospital, Osaka, Japan
7 Mitsubishi Tanabe Pharma Corporation, Osaka, Japan

Yukihito Higashi (yhigashi@hiroshima-u.ac.jp)
Tetsuro Miyata (tmiyata-tky@umin.ac.jp)
Hiroshi Shigematsu (h-shigematsu@iuhw.ac.jp)
Hideki Origasa (horigasa@las.u-toyama.ac.jp)
Masatoshi Fujita (mfujita@kuhp.kyoto-u.ac.jp)
Hiroshi Matsuo (hiro-matu@fan.hi-ho.ne.jp)
Hiroaki Naritomi (naritomi@kyowakai.com)
Hiroaki Matsuda (Matsuda.Hiroaki@mk.mt-pharma.co.jp)
Masahide Nakajima (Nakajima.Masahide@mh.mt-pharma.co.jp)
Hideto Awano (Awano.Hideto@ma.mt-pharma.co.jp)

Corresponding author:
Yukihito Higashi, MD, Department of Cardiovascular Regeneration and Medicine, Research Institute for Radiation Biology and Medicine, Hiroshima University, 1-2-3 Kasumi, Minami-ku, Hiroshima, Japan (yhigashi@hiroshima-u.ac.jp)

Supplementary Table 1. Number of Recorded Events for Individual Cardiovascular, Cerebrovascular, and Peripheral Vascular Endpoints (RWP)

	Reported Events[a]	SEASON Events[b]	
		No. of Events	No. of Patients With Events
Cerebrovascular Event			
Cerebral infarction	152	133	128
Intracerebral hemorrhage	28	24	24
Subarachnoid hemorrhage	10	9	9
Transient ischemic attack	27	16	16
Cardiovascular Event			
Myocardial infarction	64	49	48
Unstable angina	79	49	48
Heart failure	181	125	117
Peripheral vascular event			
Amputation	96	75	70
Development of critical limb ischemia	178	39	38
Acute limb ischemia	72	55	54
New onset of end-stage renal failure	22	18	17
Acute aortic dissection	5	4	4
Rupture of abdominal aortic aneurysm	5	5	5
Acute pulmonary thromboembolism	2	1	1

Abbreviations: RWP, real-world population.
[a] All events reported by the investigators.
[b] All events assessed by the Efficacy Endpoint Review Committee and judged as vascular events.
